# Pullthrough Operation for Hirschsprung's Disease: Importance of a Circumferential (Donut) Biopsy at the Level of the Anastomosis

**DOI:** 10.1055/s-0039-1693494

**Published:** 2019-07-15

**Authors:** Susan Jehangir, Soundappan Venkatraman Sannappa Soundappan, Micheal Krivanek, Susan Arbuckle, Nicole Graf

**Affiliations:** 1 Department of Paediatric Surgery, Christian Medical College, Vellore, India; 2 Department of Paediatric Surgery, The Children's Hospital at Westmead, Sydney, New South Wales, Australia; 3 Department of Anatomical Pathology, The Children's Hospital at Westmead, Sydney, New South Wales, Australia

**Keywords:** atypical aganglionosis, Hirschsprung's disease, skip lesions, zonal aganglionosis

## Abstract

Hirschsprung's disease is characterized by the absence of ganglia in the distal colon, resulting in a functional obstruction. It is managed by excision of the aganglionic segment and anastomosis of the ganglionated bowel just above the dentate line. The level of aganglionosis is determined by performing multiple seromuscular biopsies and/or full thickness biopsy on the antimesenteric border of the bowel to determine the level of pullthrough. The transition zone is described as being irregular, and hence a doughnut biopsy is recommended so that the complete circumference can be assessed. Herein, we described a child in whom there was a selective absence of ganglion cells in 30% of the circumference of the bowel along the mesenteric border for most of the transverse colon. This case defies the known concept of neural migration in an intramural and transmesenteric fashion and emphasizes the importance of a doughnut biopsy of the pulled-down segment.

## Introduction

Hirschsprung's disease (HSCR) is a disorder of migration of neural crest cells during embryonic development characterized by the absence of ganglia in the distal colon, resulting in a functional obstruction. The principles of management involve excision of the aganglionic segment and anastomosis of the ganglionated bowel above the dentate line. The level of aganglionosis is determined by performing multiple seromuscular biopsies on the antimesenteric border of the bowel. This practice is substantiated by Coventry's theory that vagal neural crest cells colonize the gut by intra- and extramural migration. 1 The intramural progression is from cranial to caudal, whereas the extramural migration occurs along the mesentery and circumferentially innervates the bowel from the mesenteric side. 1 Some authors also recommend the presence of ganglion cells and the absence of hypertrophies nerves on a full-thickness biopsy to be adequate to determine the level of pullthrough. 2 Herein, we describe a child in whom this practice would have failed because of the selective absence of ganglion cells in 30% of the circumference of the bowel along the mesenteric border for most of the transverse colon. To the best of our knowledge, this is the first report of such an occurrence and has significant diagnostic and therapeutic implications.

## Case Report

**Fig. 1:**
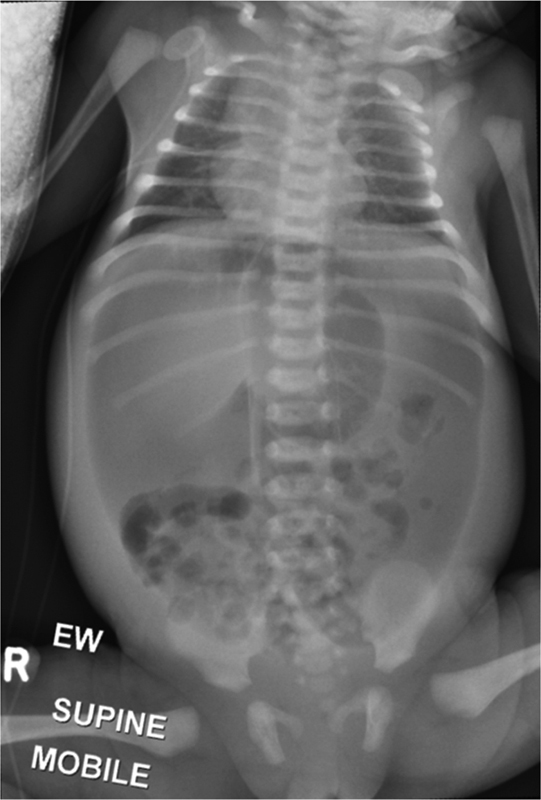
Supine plain abdominal film with evidence of free air in the peritoneal cavity.

**Fig. 2:**
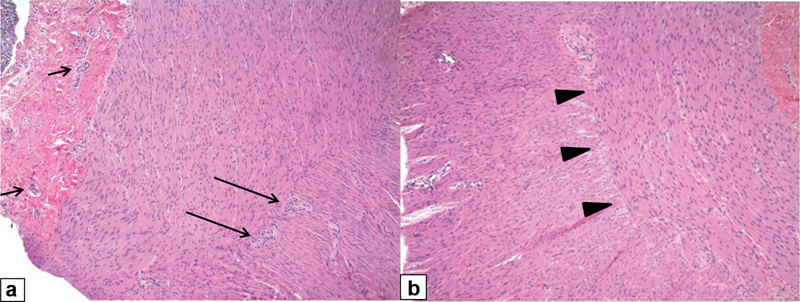
Histopathology of the doughnut showing ( **a** ) the presence of ganglion cells in 70% of the circumference on the antimesenteric side ( *long arrows* indicate the myenteric plexus, *short arrows* indicate the submucosal plexus) and ( **b** ) the absence of ganglion cells in 30% of the circumference on the mesenteric side ( *arrowheads* indicate the aganglionic myenteric plexus).

**Fig. 3:**
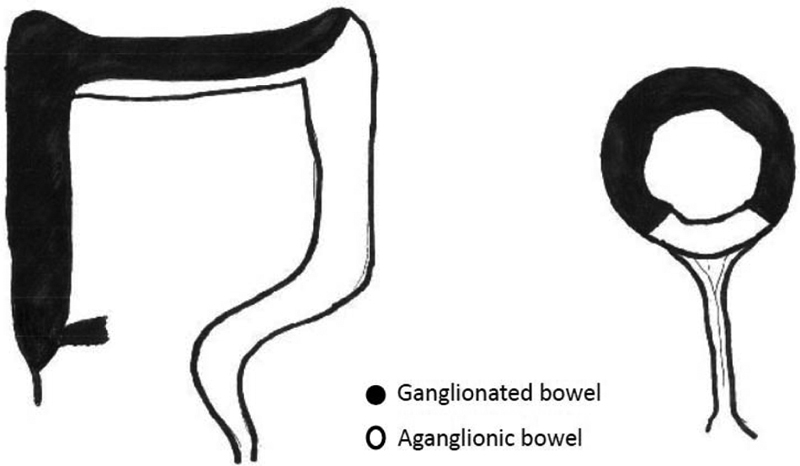
Pictorial representation of the pattern of aganglionosis.

A boy baby born at 37 weeks of gestation was transferred for specialist care for abdominal distension and delayed passage of meconium at 36 hours of life with a significant maternal family history of HSCR. Plain abdominal X-ray showed free air ( Fig. 1 ). He underwent emergency laparotomy with repair of a perforation at the splenic flexure, ileostomy, multiple seromuscular biopsies of the bowel, a rectal washout, and full-thickness rectal biopsy. The biopsies showed the absence of ganglion cells distal to the perforation at the splenic flexure and ganglion cells present at and proximal to the perforation. He underwent a pullthrough at 5 months of age. Frozen section of the proximal doughnut showed ganglion cells and no hypertrophied nerves, but some parts of the circumference were difficult to assess. It was decided to bring the splenic flexure down to complete the pullthrough. The final biopsy completed 4 days later, however, found absent ganglion cells in 30% of the circumference of the doughnut on the mesenteric side ( Fig. 2 ). Findings were discussed with the family, and they wished for normal bowel to be pulled through. A redo pullthrough was performed 9 days after the original operation. Intraoperative biopsies at the revision pullthrough showed the absence of ganglion cells in 30% of the circumference along the mesenteric border in the entire transverse colon ( Fig. 3 ). The hepatic flexure was tumbled down. The postoperative recovery was uneventful, and the child remains well at follow-up except for frequent stooling. The genetic work-up is pending.

## Discussion

HSCR is thought to be the result of arrested enteric neural crest cell (ENCC) migration, which occurs rostrocaudally. The level of arrest determines the length of the aganglionic segment. Rare variations in the pattern of aganglionosis are well described and are categorized into “skip” lesions, in which there is a segment of ganglionated bowel surrounded proximally and distally by aganglionosis, occurring most commonly with total colonic aganglionosis. 3 Another variant described is the “zonal” aganglionosis, in which a segment of aganglionic bowel is surrounded by normal ganglionated bowel. The concept of “double zonal aganglionosis” is applied to HSCR with a skip lesion. 3 The presence of skip lesions is explained by the mesenteric migration of ENCCs that colonize a segment of bowel within the aganglionic segment ahead of the wavefront of distally migrating ENCCs. However, the pattern of aganglionosis in our patient is unique and defies the current understanding of the embryogenesis of HSCR.

ENCC precursors originate in the vagal region (somites 1–7) and to a lesser extent from the thoracic and sacral regions of the neural tube. 3 They enter the gut mesenchyme by the third week of gestation and migrate in a craniocaudal pattern, proliferate, differentiate, and colonize the gut completely by the eighth week of gestation, forming the network of the enteric nervous system. This theory, however, does not explain the defect in our patient. We hypothesize the following various mechanisms that could cause the pattern of aganglionosis in our patient.

- Failure of transmesenteric migration: in a mouse model, Druckenbrod and Epstein studied the transmesenteric migration of ENCCs from the midgut to the hindgut during the time these organs are transiently juxtaposed. 4 This migratory process requires glial cell derived neurotrophic factor (GDNF) signaling. In the absence of this transmesenteric migration, the intramural migration may continue along the antimesenteric border and cause a defect such as in our patient. The failure of transmesenteric migration could be because of a lack of juxtaposition, inadequate numbers of ENCCs due to a preexisting genetic defect, or defects in GDNF signaling.
- Delayed migration: ENCC colonization has been shown to be a timed process. 5 Furthermore, ENCC migration is enhanced when the cells are in contact with each other along the wavefront. Isolated ENCCs do not migrate as quickly or as directionally as chains of cells. Mutations in L1 cell adhesion molecule (L1CAM), a protein that maintains such cell–cell contacts, reduce ENCC contact and can retard ENCC migration. 5 The microenvironment may no longer be permissive to colonization by the time they arrive.
- Genetic factors provide the framework for patterning and morphogenesis. However, it is now known that maternal and placental factors such as hypoxia, inflammation, drug intake, and nutrition can affect the survival of ENCC. 6 This concept shed new light on the existing theories of aganglionosis. The area of aganglionosis in our patient involved the watershed area of the intestine. Did a hostile environment due to transient hypoxia, inflammation, or nutrient deficiency destroy the colonized ENCCs and cause the defect?

The pattern of aganglionosis in the transition zone is unusual, and a proper doughnut biopsy will avoid a transition zone pullthrough. 2

## Notes

New Insights and the Importance for the Pediatric Surgeon A careful doughnut biopsy of the pullthrough segment is essential to avoid missing unusual patterns of aganglionosis
